# Seeing Colors: Cultural and Environmental Influences on Episodic Memory

**DOI:** 10.1177/2041669517750161

**Published:** 2017-12-20

**Authors:** Kimele Persaud, Pernille Hemmer, Celeste Kidd, Steven Piantadosi

**Affiliations:** Department of Psychology, Rutgers University, Piscataway, NJ, USA; Department of Brain and Cognitive Sciences, 6927 University of Rochester, Rochester, NY, USA

**Keywords:** cognition, color, memory, perception

## Abstract

Expectations learned from our perceptual experiences, culture, and language can shape how we perceive, interact with, and remember features of the past. Here, we questioned whether environment also plays a role. We tested recognition memory for color in Bolivia’s indigenous Tsimanè people, who experience a different color environment than standard U.S. populations. We found that memory regressed differently between the groups, lending credence to the idea that environmental variations engender differences in expectations, and in turn perceptual memory for color.

Principles of rationality (Anderson, 1990) assume that people optimize cognitive behaviors relative to the demands of the environment and costs to the cognitive system. So, can our environment really have us *seeing* red? Maybe, if you were a regular at Rutgers University where the school colors, Scarlet Red and Black, adorn every building and sign post. The ubiquity of these colors, or any statistical regularity in our environment for that matter, may impact how we perceive and communicate and might provide useful cues when recalling events from long-term memory. For example, recalling the color of the shirt you wore at Saturday’s Rutgers Football game might be based not only on vague recollections but also biased by your environment. Now, what if you are not a regular at Rutgers but instead at some other university? Would this difference in environment have you seeing (and recalling) different shades of red, that is, could differences in environmental structure, such as geographic locations and cultural profiles, differentially influence perceptual expectations and thus memory?

Despite language having been cited as the main culprit for differences, for example, in color memory across cultures (Roberson, Davidoff, Davies, & Shapiro, 2005), we found ourselves wondering whether the environment may also play a role in promoting such differences, consistent with the principles of rationality.

By happenstance, two of the authors were on their way to Bolivia to work with a native population, the Tsimanè, in an arguably very different physical environment from the United States generally—the lowland rainforests of Bolivia—providing a rare opportunity to explore these questions. The Tsimanè live a pseudo *hunter-gatherer* lifestyle and have little contact with industrialized communities in Bolivia. There are well-known group differences in color naming and short-term memory across populations (Roberson et al., 2005), for example, there is evidence showing that hunter-gatherer-like communities, similar to the Tsimanè, have three to five lexical color categories (Lindsey, Brown, Brainard, & Apicella, 2015). In Tsimanè language, color words are highly variable and, as is the case in other languages, when there is not a label for the color, it is labeled with a description—for example, yellow might be called *color-of-the-cuchicuciyeisi-tree* (i.e., the cuchi [*Astronium urundeuva*] tree native to Bolivia). Whether due to variability in education or lack of communicative need (i.e., low prevalence of some colors in the environment), some people know color words, while others do not.

The other two authors had previously assessed episodic memory in the domain of color, as well as bidirectional categorical expectations (assignment of label to hue and hue to label), in a U.S. population (Persaud & Hemmer, 2014). This work showed a regression to the mean effect, where studied values within a perceptual category were biased toward the category mean of seven classic basic color terms. The regression to the mean effect is evidence of the influence of expectations on memory (Bae, Olkonnen, Allred, & Flombaum, 2015; Hemmer & Steyvers, 2009a, 2009b; Huttenlocher, Hedges, & Vevea, 2000). There was a direct match between category expectations in the bidirectional tasks and the seven categories to which memory regressed.

Environmental and cultural (e.g., language) differences between the Tsimanè and the U.S. populations are pronounced and provided an ideal setting for assessing possible differences in regression to the mean effects in memory. With room for just one study in the field trip, we focused on assessing episodic memory in order to learn the underlying color categories of the Tsimanè. We once again base our assumptions on the principle of rationality, and a Bayesian model of memory, which posits that two streams of information—noisy episodic memory and expectation for the environment—are necessarily integrated to produce recall (Hemmer & Steyvers, 2009b). Given this framework and our results from the U.S. population, we can work backward to infer category expectations from memory performance.

The Tsimanè participants (*N* = 23) completed a paper based six-alternative forced choice recognition task for 24 unique color-shape pairings where participants only needed to point to respond (Figure 1(a) and (b)). The 24 colors varied in hue by a minimum of 5 units (on a total range of 239) and were randomly selected from 7 color categories, with samples proportional to the size of the color category. Saturation and luminance were held constant at 100% and 50%, respectively. Participants studied a single colored shape for 1 second and were immediately asked to recognize the studied color using a six-alternative forced choice set. Participants had as much time as needed, but most responded immediately. Responses were recorded in a booklet. Trial order was randomized between participants. The task was administered in communal classrooms with onlookers, and responses required two layers of translation (i.e., from English to Spanish and then from Spanish to the Tsimanè language).

**Figure 1.**
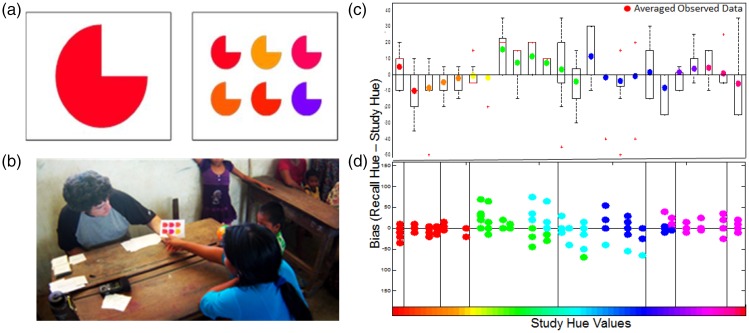
(a) Sample stimuli: study (left)/test (right). (b) Tsimanè participating in study. (c) Y-axis: Mean recognition bias (data points) for each studied hue value and response ranges (boxplots). *X*-axis: 24 studied colors. (d) K-means cluster partition color coded based on the classification results.

Memory bias (recalled hue value minus studied hue value—e.g., studying a hue of 5 and recalling a hue value of 15 results in a bias of 10) regressed toward the mean of some classic color categories, but not others (Figure 1(c)). In contrast to the U.S. group, the Tsimanè segregated blue into two categories and combined other categories, resulting in five inferred categories: red/orange/yellow, green, light blue, dark blue, and purple/pink. An unsupervised k-means cluster analysis on the remembered hue values (Figure 1(d)) was conducted in Matlab with 10 iterations on four cluster sizes and confirmed by the Calinski Harabasz criterion. This analysis showed the greatest agreement for this five category partition. While the splitting of the blue category is reminiscent of findings from Russian speakers (Winawer et al., 2007), the more interesting finding is the unsplit *warm* category (i.e., red, orange, and yellow hues), which is consistent with the findings of Gibson et al. (2017) that the top free-choice colors of the Tsimanè do not include orange or pink.

While the bias patterns observed in the Tsimanè, relative to the bias in the U.S. population, might be related to the underdevelopment of some categories or low frequency in their language, it could also be due to low environmental incidence, and thus little communicative need of certain terms. In short, we proffer the idea that it is not just language that promotes differences in color memory across cultures but it could also be environmental structure and color prevalence. If color memory reflects rational inference under uncertainty, we should expect to see bias patterns that reflect either color language, or environmentally determined priors, or both. A general prediction shared by all these possibilities is that participants from a population speaking a language other than English and living in an environment other than the United States should exhibit bias patterns in color memory that differ from those of English speakers in the United States. Here, we have shown that this is true, given new data from a culture not previously studied in this context. We leave for future work the question of what amount of this cross-cultural difference in color memory bias is due to language, versus environment, versus other possible influences, and we note that a strength of the Bayesian account is that it is not restricted to a single source of influence on memory, but could in principle accommodate a mixture of such influences.

## Authors’ Note

Data were previously presented at the annual meeting of the Cognitive Science Society, 2014.
